# 925. Burn Prevention in the Home: Community Smoke Alarm Installation

**DOI:** 10.1093/jbcr/irag033.465

**Published:** 2026-04-06

**Authors:** Tiffany N Lord, Michael J Feldman, Christy Carneal, Elizabeth Allison Bowers

**Affiliations:** Evans-Haynes Burn Center at VCU Health, Richmond, VA; Virginia Commonwealth University, Richmond, VA; Longwood University, Petersburg, VA; Evans-Haynes Burn Center at VCU Health, Richmond, VA

## Abstract

**Introduction:**

Home fires are the nation’s most frequent disaster. Seven people die every day as a result from home fires, costing $7 billion in property damage annually. Working smoke alarms can cut the risk of death by half. In response, the American Red Cross began the Home Fire Campaign in 2014. The campaign’s major initiative is Sound the Alarm, which organizes volunteers in a door-to-door effort, offering free smoke alarm installation and burn prevention education in at-risk neighborhoods. As a result of the national effort, 2479 lives have been saved, 3 155 254 smoke alarms have been installed, and 3 405 211 community members have been served. To increase our own community-based injury prevention, our burn center has partnered with the American Red Cross to conduct regular Sound the Alarm campaigns in our surrounding communities.

**Methods:**

Three Sound the Alarm campaigns were conducted over a two-year period. Heat mapping of surrounding communities identified zip codes with the highest rates of residential structure fires. Neighborhoods were canvased the week prior to the event, leaving flyers with residents notifying them of the upcoming campaign. On the day of the event, teams of volunteers went door-to-door installing free smoke alarms. During smoke alarm installation, in-home burn prevention education was provided. Each household was left with literature regarding developing a home fire escape plan and seasonal burn prevention education.

**Results:**

309 smoke alarms were installed in 189 homes. Fire and burn prevention education was delivered to 489 community members. Interviews were conducted with staff after each event. Burn center personnel expressed that the effort was a positive way to engage with the community regarding burn prevention. Several staff reported that they would like this to be a reoccurring event. Interviews with household members revealed eager participation. Several families reported stories of residential structure fires in their neighborhoods, which left them concerned for their own safety.

**Conclusions:**

Participation in Sound the Alarm campaigns has allowed burn center staff the ability to participate in impactful community burn prevention and education. Community members expressed gratitude for the Sound the Alarm campaign.

**Applicability of Research to Practice:**

The American Red Cross’s Home Fire Campaign and Sound the Alarm is a national effort. Burn centers from across the country can contribute to the initiative with events in their own communities.

**Funding for the study:**

This initiative is funded at the national and local level through corporate partner donations and grants. Items are also purchased through the American Red Cross’ regional budget.

